# Effectiveness and Safety of Intensive Triplet Chemotherapy Plus Bevacizumab, FIr-B/FOx, in Young-Elderly Metastatic Colorectal Cancer Patients

**DOI:** 10.1155/2013/143273

**Published:** 2013-11-06

**Authors:** Gemma Bruera, Katia Cannita, Aldo Victor Giordano, Roberto Vicentini, Corrado Ficorella, Enrico Ricevuto

**Affiliations:** ^1^Medical Oncology, S. Salvatore Hospital, University of L'Aquila, 67100 L'Aquila, Italy; ^2^Department of Biotechnological and Applied Clinical Sciences, University of L'Aquila, 67100 L'Aquila, Italy; ^3^Radiology, S. Salvatore Hospital, 67100 L'Aquila, Italy; ^4^Hepatobiliar-Pancreatic Surgery, S. Salvatore Hospital, 67100 L'Aquila, Italy

## Abstract

Four-drug regimens, such as FIr-B/FOx schedule, can improve efficacy of first-line treatment of metastatic colorectal cancer (MCRC) patients. The present study specifically evaluates feasibility of FIr-B/FOx first-line intensive regimen in fit young-elderly MCRC patients, representing approximately 40% of overall MCRC patients. Activity, efficacy, and safety were equivalent to overall MCRC patients, not significantly different according to *KRAS* genotype. Clinical outcome was significantly prolonged in liver-limited compared to other/multiple metastatic disease. Safety evaluation of the individual young-elderly patient showed that limiting toxicity syndromes (LTS) in multiple sites were significantly increased, compared to LTS in single site, with respect to non-elderly patients.

## 1. Introduction

Clinical management of MCRC is faced with different options and lines of treatment according to patients' fitness, extension of metastatic disease, and *KRAS* genotype [1–6]. First line triplet regimens of chemotherapeutic drugs, or doublet associated to bevacizumab (BEV) or cetuximab, reported in phase III trials objective response rate (ORR) 39%–68%, progression-free survival (PFS) 7.2–10.6 months, and overall survival (OS) 19.9–26.1 months [2, 4, 6–8]. More intensive triplet chemotherapy plus targeted agents can further achieve ORR 82%, liver metastasectomies 26%, PFS 12 months, OS 28 months [1–5]. In liver-limited (L-L) disease, metastasectomies were 54%, and clinical outcome was significantly improved, particularly in *KRAS* wild-type patients [3, 5].

Older patients are usually underrepresented in clinical trials, despite the increased incidence with age, and often undertreated in clinical practice. Retrospective studies showed similar safety and efficacy in fit elderly compared to younger patients [9–11]. Elderly patients require a decision-making process including functional, nutritional, and co-morbidity status to discriminate fitness and tailor medical treatment [12]. Fit patients ≥70 years benefit from 5-fluorouracil (5-FU) as younger patients: ORR 23.9%, PFS 5.5 months, and OS 10.8 months [13]. A retrospective review and a pooled analysis reported no different activity and efficacy [14, 15]. The same benefit was reported from irinotecan (CPT-11) containing chemotherapy in fit older ≥70 years [16]; age was not an independent prognostic factor for OS [17]. The significantly improved relative benefit of FOLFOX did not differ by age [18]. In the OPTIMOX1 trial, ORR 59%, median PFS 9.0 months, and median OS 20.7 months were comparable in old-elderly patients [19]. In the FOCUS2 trial, specifically designed to evaluate first line reduced-dose (80%) of 5-FU or capecitabine with or without oxaliplatin (OXP), in old-elderly and/or frail patients, addition of OXP significantly improved ORR, and trendly PFS, but not OS [20]. Treatment efficacy was consistent across subgroups, including age, when BEV was combined with CPT-11-based therapy [21]. In fit elderly patients, addition of BEV to 5-FU based chemotherapy significantly prolonged PFS (9.2-9.3 months) and OS (17.4–19.3 months) [22, 23]. In BRiTE and BEAT studies, no different PFS was observed; median OS decreased with age [24, 25]. No impact on PFS and OS was observed by age and/or comorbidities in patients treated with FOLFOX or FOLFIRI added or not to cetuximab [26]. Addition of panitumumab to FOLFOX showed no clear benefit in PFS in elderly and performance status 2 patients [27]. 

 In the randomized phase III trial comparing FOLFOXIRI with FOLFIRI, age was not a significant factor for activity and efficacy; elderly patients showed median OS 16.9 and 19.9 months with FOLFIRI or FOLFOXIRI, respectively [28, 29]. ORR was significantly lower in older patients treated with FOLFOXIRI [29]; no differences were reported in PFS and OS. Patients underwent metastasectomies without increased morbidity or mortality, irrespective of age.

 Here, we report a retrospective analysis evaluating activity, efficacy, and safety of first-line FIr-B/FOx intensive regimen and the prognostic value of extension of metastatic disease [4, 5] in fit young-elderly MCRC patients enrolled in a previously reported phase II study [1] and in the expanded clinical program proposing first-line FIr-B/FOx treatment. 

## 2. Materials and Methods

### 2.1. Patient Eligibility

Present retrospective analysis evaluated consecutive young-elderly patients 65 to 75 years enrolled in a previously reported phase II study [1] and in the expanded clinical program proposing first-line FIr-B/FOx treatment. Patients who were eligible were with histologically confirmed diagnosis of measurable MCRC, performance status ≤2, adequate hematological, renal, and hepatic functions, and life expectancy >3 months. Patients were not eligible if they showed uncontrolled severe diseases; cardiovascular disease (uncontrolled hypertension, uncontrolled arrhythmia, and ischemic cardiac diseases in the last year); thromboembolic disease, coagulopathy, and preexisting bleeding diatheses; proteinuria >1 g/24 h urine; surgery within the previous 28 days before. Cumulative Index Rating Scale (CIRS) was used to evaluate the comorbidity status, and only patients with primary and intermediate CIRS stage were enrolled [12]. Primary CIRS stage consisted of independent Instrumental Activity of Daily Living (IADL) and absent or mild grade comorbidities; intermediate CIRS stage consisted of dependent or independent IADL and less than 3 mild or moderate grade comorbidities. Patients with secondary CIRS stage, consisting of more than 3 comorbidities or a severe comorbidity, with or without dependent IADL, were not enrolled. The study was approved by the Local Ethical Committee (Comitato Etico, Azienda Sanitaria Locale n.4 L'Aquila, Regione Abruzzo, Italia) and conducted in accordance with the Declaration of Helsinki. All patients provided written, informed consent.

## 3. Methods

### 3.1. Schedule

FIr-B/FOx regimen consisted of weekly timed flat-infusion/5-fluorouracil (TFI 5-FU) [30, 31], associated to weekly alternating CPT-11/BEV or OXP [1]: TFI 5-FU (Fluorouracil Teva; Teva Italia, Milan, Italy), 900 mg/m^2^/day, over 12 h (from 10:00 pm to 10:00 am), days 1, 2, 8, 9, 15, 16, 22, and 23; CPT-11 (Campto; Pfizer, Latina, Italy), 160 mg/m^2^, days 1, 15; BEV (Avastin; Roche, Welwyn Garden City, UK), 5 mg/kg, days 1, 15; l-OXP (Eloxatin; Sanofi-Aventis, Milan, Italy), 80 mg/m^2^, days 8, 22; cycles every 4 weeks.

### 3.2. Mutational Analysis

Genetic analyses were performed on paraffin-embedded tissue blocks from the primary tumor and/or metastases, as previously reported [5]. Genotype status was assessed for *KRAS* codon 12, 13, and *BRAF* c.1799 T>A (V600E) mutations by SNaPshot multiplex assay in 17 samples, as elsewhere reported [32, 33]. Briefly, *KRAS* exon 2 and *BRAF* exon 15 were simultaneously PCR-amplified and analyzed for *KRAS* c.34G, c.35G, c.37G, c.38G, and *BRAF* c.1799T mutations using the ABI PRISM SNaPshot Multiplex kit (Applied Biosystems, Foster City, CA, USA). *KRAS* exon 2 direct sequencing was performed using the Big Dye V3.1 Terminator Kit (Applied Biosystems, Foster City, CA, USA). Labelled products were separated in ABI Prism 3130*xl* Genetic Analyzer (Applied Biosystems, Foster City, CA, USA) and analysed using the GeneMapper Analysis Software version 4.0 (Applied Biosystems, Foster City, CA, USA).

### 3.3. Study Design

Response was evaluated by computed tomography scan; positron emission tomography was added based on investigators' assessment. Follow-up was scheduled every three months up to progression or death. Resectability, defined according to reported categories [3], was evaluated in patients with L-L metastases every three cycles by a multidisciplinary team, consisting of a medical oncologist, liver surgeon, and radiologist, and recommended >4 weeks after BEV discontinuation. Liver metastasectomies were defined as R0, if radical surgery, R1, if radioablation was added. 

Toxicity was registered according to the National Cancer Institute Common Toxicity Criteria (version 3.0). Limiting toxicity (LT) was defined as grade 3-4 non-hematological toxicity, grade 4 hematologic toxicity, febrile neutropenia, or any toxicity determining >2 weeks treatment delay. To discriminate individual safety, limiting toxicity syndromes (LTS), consisting of at least an LT associated or not to other limiting or G2 toxicities, were evaluated, as previously reported [1]. LTS were classified as limiting toxicity syndromes single site (LTS-ss), characterized only by the LT, and limiting toxicity syndromes multiple sites (LTS-ms), ≥2 LTs or an LT associated to other, at least G2, non-limiting toxicities. Chi-square test was used to compared the rates of LTS-ms and LTS-ss [34].

 Clinical criteria of activity and efficacy were ORR, PFS and OS. ORR was evaluated according to RECIST criteria [35]; pathologic complete response was defined as absence of residual cancer cells in surgically resected specimens. PFS and OS were evaluated using the Kaplan-Meier method [36]. PFS was defined as the length of time from the beginning of treatment and disease progression or death (resulting from any cause) or to the last contact and OS as the length of time between the beginning of treatment and death or to last contact. Log-rank test was used to compare PFS and OS according to *KRAS* genotype and metastatic extension, L-L versus other or multiple metastatic (O/MM) [37]. 

## 4. Results

### 4.1. Patient Demographics

From March 2006 to November 2011, 28 young-elderly patients were enrolled among overall MCRC patients (42%); 26 (93%) were evaluable for *KRAS* genotype, 13 wild-type, and 13 mutant (Table 1). Patients fitting for intensive FIr-B/FOx treatment, according to inclusion criteria, represented 44% of consecutively observed MCRC patients, and this rate was equivalent for fit young-elderly patients. Demographic and baseline features were representative of the overall phase II study population: WHO Performance Status 0, 25 (89%), CIRS primary/intermediate, 2/26. Liver metastases affected 17 patients (61%), L-L 8 patients (29%), and O/MM 20 patients (79%). *KRAS *mutations were not differently represented with respect to overall MCRC patients (see, Supplementary material Table 1 at http://dx.doi.org/10.1155/2013/143273, which describes *KRAS* mutations): c.35 G>A (G12D), 8 (30.7%); c.35 G>T (G12V), 3 (11.5%); c.35 G>C (G12A), 1; c.38 G>A (G13D), 1. Seventeen tumoral samples (65%) were also analyzed for *BRAF*, and no mutation was detected. 

### 4.2. Activity and Efficacy

**Figure 1:**
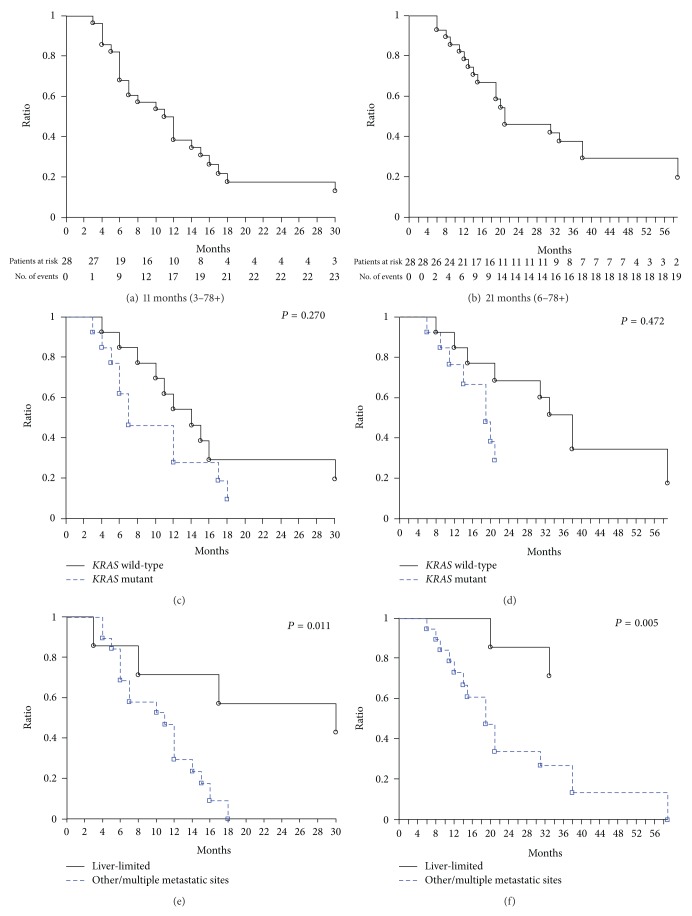
Legend Kaplan-Meier survival estimate: (a) overall population, progression-free survival; (b) overall population, overall survival; (c) overall population *KRAS* wild-type versus *KRAS* mutant, progression-free survival; (d) overall population *KRAS* wild-type versus *KRAS* mutant, overall survival; (e) liver-limited versus other/multiple metastatic sites, progression-free survival; (f) liver-limited versus other/multiple metastatic sites, overall survival.

In the intent-to-treat analysis of 28 evaluable young-elderly patients, ORR was 79% (*α* 0.05, CI ± 15) (Table 2). We observed 22 objective responses: 19 partial (68%) and 3 clinical complete (CR 11%), 1 stable (4%), and 5 progressive diseases (18%). Disease control rate was 82% (*α* 0.05, CI ± 14). After a median follow-up of 17 months, median PFS was 11 months (3–78+). Median OS was 21 months (6–78+) (Figures 1(a) and 1(b)). Liver metastasectomies were performed in 5 pts (18%): 3 out of 8 L-L pts (37.5%). In one *KRAS* wild-type patient with single liver associated with lung metastases, double metastatic resections were performed. In one *KRAS* mutant patient with single liver associated with single lung metastasis, liver metastatic resection was performed, and a clinical CR of lung metastasis was obtained. Overall, R0 liver resections were 4 (80%) and R1 resection was 1 (20%). No surgery-related complications were reported. Overall, 3 clinical plus 2 pathologic CRs were reported (18%): 2 clinical CR in *KRAS* wild-type patients and 3 in *KRAS* mutant patients (1 clinical CR and 2 pathological CR). Pathologic CRs were obtained in 2 *KRAS* mutant patients, harboring c.35 G>T and c.35 G>A mutations, with multiple L-L metastases and single liver plus single lung metastases, respectively, who obtained a clinical partial response after treatment. One patient progressed at 17 months; 4 patients were progression-free at 78, 69, 49, and 10 months. Overall, 16 patients (57%) received a second line treatment: FIr-B/FOx rechallenge, 3 (19%); cetuximab-containing treatment, 9 (56%); BEV-containing, 1 (6%); panitumumab, 1 (6%); capecitabine, 1 (6%); surgery, 1 (6%). Most *KRAS* wild-type patients received a second line anti-EGFR-containing treatment (7 out of 9, 78%); BEV-containing, 1 (11%); surgery, 1 (11%). Seven patients (25%) received a third line treatment: cetuximab-containing treatment, 2 (28.5%); panitumumab, 3 (43%); capecitabine, 2 (28.5%). Three patients (11%) received a fourth line treatment: CPT-11, 1 (33%); raltitrexed, 1 (33%); capecitabine, 1 (33%). Three patients (11%) received treatment beyond the fourth line: fifth line cetuximab-containing treatment, 1 (33%), raltitrexed, 1 (33%); sixth line capecitabine, 1 (33%).

Among 13 *KRAS* wild-type patients, ORR was 92% (*α* 0.05, CI ± 15) (Table 2). We observed 12 objective responses: 10 partial (77%) and 2 CR (15%) and 1 progressive disease (8%). Liver metastasectomies were performed in 3 patients (23%), 2 out of 4 L-L (50%). Median PFS was 14 months (4–78+ months). Median OS was 38 months (8–78+ months). Among the 9 *KRAS*/*BRAF* wild-type patients, ORR was 89% (*α* 0.05, CI ± 22), median PFS was 11 months (4–49+ months), and median OS was 23 months (8–59 months). Among 13 *KRAS* mutant patients, ORR was 77% (*α* 0.05, CI ± 24). We observed 10 objective responses: 9 partial (69%) and 1 CR (8%), 1 stable (8%), and 2 progressive diseases (15%). Disease control rate was 85% (*α* 0.05, CI ± 20). Liver metastasectomies were performed in 2 patients (15%) out of 8 L-L (20%). Median PFS was 7 months (3–69+ months). Median OS was 19 months (6–69+ months). *KRAS *wild-type compared with mutant patients did not show significantly different PFS nor OS (Figures 1(c) and 1(d)). 

### 4.3. Dose-Intensity and Toxicity

Median number of cycles per patient was 5 (range 2–9). Median received dose intensities (rDI) per cycle were equivalent to overall patients: 5-FU 1440 (480–1800) mg/m^2^/w, 80%; CPT-11 64 (25–80) mg/m^2^/w, 80%; l-OXP 32 (8–40) mg/m^2^/w, 80%; BEV 2 (1–2.5) mg/kg/w, 80% (see Supplementary material, Table 2, which describes rDI). 

One patient (3.5%) discontinued FIr-B/FOx treatment due to limiting toxicity (grade 3 diarrhea). G3-4 toxicities, by patients, in 134 cycles, were (Table 3) diarrhea, 6 (21%); stomatitis/mucositis, 3 (11%); asthenia, 3 (11%); and neutropenia 3 (11%). The prevalent toxicity was diarrhea, G2-G3 in 14 patients (50%), similar to non-elderly [1]. G2 toxicities were nausea 11 (39%), vomiting 3 (11%), diarrhea 8 (29%), asthenia 11 (39%), neurotoxicity 4 (14%), hypertension 3 (11%), and neutropenia 11 (39%). No cases of thrombosis, hemorrhage/bleeding, cardiac or cerebrovascular ischemia, G4 neutropenia, febrile neutropenia, severe thrombocytopenia, or toxic deaths were observed. LTS were observed in 13 out of 28 young-elderly patients (46%) (Table 4): LTS-ms, 11 pts (39%) and LTS-ss, 2 pts (7%). LTS-ms were characterized by: LT associated to other, at least G2, non-limiting toxicities, 9 pts (32%); and ≥2 LTs, 2 pts (7%). LTS were significantly represented by LTS-ms compared to LTS-ss (chi-square 3.832, *P* = 0.05), with respect to non-elderly patients. LTS were (see Supplementary material, Table 3, which describes toxicities characterizing LTS in individual patients) G2-3 diarrhea-associated, 9 patients (69.2%), 8 LTS-ms and 1 LTS-ss; G3 mucositis associated with G3 erythema, 1; G3 stomatitis/mucositis and G2 asthenia, 1; G2 neutropenia for >2 weeks with G2 nausea, 1; and G3 asthenia, 1. 

### 4.4. Activity and Efficacy according to *KRAS* Genotype and Extension of Metastatic Disease

Among 7 L-L patients, ORR was 86% (*α* 0.05, CI ± 28) (see Supplementary material, Table 4, which describes activity, efficacy, and effectiveness of FIr-B/FOx regimen according to *KRAS* genotype and extension of metastatic disease); 3 performed liver metastasectomies (43%) and 3 cCRs (43%) in patients who did not undergo liver surgery and showed PFS of 78+, 69+, and 49+ months; median PFS was 30 months (3–78+ months); median OS was not reached (20–78+ months) at a median follow-up of 49 months. Among 19 evaluable O/MM patients, ORR was 84% (*α* 0.05, CI ± 17); median PFS was 11 months (4–18 months); median OS was 19 months (6–59 months). Overall, clinical outcome (PFS and OS) in L-L compared to O/MM patients was significantly different (Figures 1(e) and 1(f)): among *KRAS* wild-type (see Supplementary material, Figure 1(a), which reports PFS and OS of *KRAS* wild-type patients, L-L versus O/MM), *P* 0.058 for PFS and *P* 0.035 for OS; among *KRAS* mutant (see Supplementary material, Figure 1(b), which reports PFS and OS of *KRAS* mutant patients, L-L versus O/MM), not significantly different. 

## 5. Discussion

First line medical treatment of MCRC patients consists of triplet regimens including chemotherapeutic drugs, or doublets plus BEV, or doublets plus EGFR-inhibitors in *KRAS* wild-type patients, showing ORR 39%–68%, PFS 7.2–10.6 months, and OS 19.9–26.1 months [2, 4, 7, 8]. Triplet FOLFOXIRI regimen gained ORR 60%, PFS 9.8 months, and OS 23.4 months, and recently showed 5 years-PFS 5% and 5 years-OS 15% [7]. More intensive regimens, consisting of triplet chemotherapy plus targeted agents, can further increase activity, efficacy, and effectiveness of liver metastasectomies [1, 38, 39]. Phase II studies, by Masi et al. [38], and by our group [1], proposed BEV addition to triplet chemotherapy, according to FOLFOXIRI/BEV or FIr-B/FOx schedules, reaching ORR 77% and 82%, liver metastasectomies 40% and 54% in L-L disease, median PFS 13.1 and 12 months, and median OS 30.9 and 28 months. Present retrospective analysis showed that young-elderly patients represented 42% of MCRC patients treated with FIr-B/FOx intensive regimen, mainly characterised by performance status 0 (89%) and intermediate CIRS (93%) stage and confirmed high activity and efficacy (ORR 79%, PFS 11 months, and OS 21 months), as reported in overall MCRC patients [1]. 

 Retrospective analysis of doublets CPT-11, or OXP, associated to 5-FU or capecitabine in older patients reported ORR 18–59.4%, PFS 4.9–10.0 months, and OS 8.5–20.7 months [13–20, 29, 40]. The addition of BEV to 5-FU-based chemotherapy in elderly patients significantly increased PFS 9.2-9.3 and OS 17.4–19.3 months [22, 23]. Triplet chemotherapy or doublet plus BEV obtained ORR 34.9-45.9%, PFS 7.9–9.3 months, and OS 17.4–20.5 months [23–25]. In the HORG-FOLFOXIRI trial, no different clinical outcome was observed in elderly patients; significantly lower PFS and OS were reported in patients with performance status 2 [28, 29]. Liver metastasectomies were reported in 1.3% and 4.2% patients treated with FOLFIRI and FOLFOXIRI, respectively, [29] and can achieve OS 43 months, not significantly different from younger patients [41]. Morbidity and/or mortality after liver surgery were significantly higher in elderly patients (8%) [42]. Our present retrospective data show that intensive FIr-B/FOx treatment of young-elderly MCRC patients, carefully selected according to comorbidity and functional status, may achieve increased activity and clinical outcome than that reported. The high activity is correlated with 18% liver resection rate, 37.5% in L-L patients, and 40% pathologic CR, without increased morbidity and/or mortality. 

 FOLFOXIRI plus BEV and FIr-B/FOx schedules may increase activity and efficacy in patients with *KRAS* wild-type and mutant genotypes [5, 38]. Median OS of patients treated with FIr-B/FOx was different in *KRAS* wild-type and mutant patients (38 months and 21 months, resp.), but not significantly different [5]. Similarly, FIr-B/FOx clinical outcome was not significantly different according to* KRAS* genotype, in young-elderly patients. Our previous reports of significantly different clinical outcome of L-L compared to multiple metastatic disease [3], particularly in *KRAS* wild-type patients, while not in *KRAS *mutant [5], were confirmed in young-elderly patients and should be prospectively verified.

FIr-B/FOx in young-elderly patients was feasible at median rDI 80%. Cumulative G3-4 toxicities were prevalently represented by diarrhea (21%), stomatitis/mucositis (11%), asthenia (11%), and neutropenia (11%). Individual LTS were reported in 46% young elderly patients, mainly including diarrhea (69.2%), and significantly more represented by LTS-ms compared to LTS-ss (chi-square 3.832, *P* = 0.05), with respect to non-elderly patients. Published studies showed that grade 3/4 toxicities were not significantly different in elderly patients treated with 5-FU or CPT-11 [14–16], slightly increased with FOLFOX [19], and significantly increased by capecitabine (40%), while not by the addition of OXP [20]. Limiting diarrhea was significantly higher with FOLFIRI and FOLFOXIRI [28, 29]. Performance status 2 was significantly associated with increased grade 3/4 neutropenia, febrile neutropenia, diarrhea, and fatigue, compared with performance status 0-1 [28, 29, 40]. In elderly patients, BEV addition to chemotherapy was significantly associated with increased arterial thromboembolism [43], while not to other adverse events [22–25]. The present retrospective, exploratory analysis in a small cohort of MCRC patients, showed that intensive FIr-B/FOx schedule is equivalently safe and feasible, without severe adverse events related to BEV, in young-elderly patients, selected by favourable performance status and functional and comorbidity status, with a rate of LTS-ms significantly increased compared to LTS-ss, with respect to non-elderly patients. Young-elderly MCRC patients suitable for FIr-B/FOx intensive treatment should be carefully selected based on comorbidity and functional status and monitored for individual safety in clinical practice. 

## 6. Conclusions

In fit young-elderly patients, FIr-B/FOx intensive regimen is safe, with toxicity characterized by LTS-ms, high activity, efficacy, and liver metastasectomies, particularly in L-L, *KRAS* wild-type, compared to O/MM. Present findings would be prospectively verified in a larger cohort of young-elderly MCRC patients.

## Supplementary Material

Among Supplementary material, Table 1 describes KRAS mutations detected; Table 2 describes received dose-intensities; Table 3 describes toxicities characterizing limiting toxicity syndromes in individual patients; Table 4 describes activity, efficacy and effectiveness of FIr-B/FOx regimen according to KRAS genotype and extension of metastatic disease; Figure 1 reports progression-free survival and overall survival of KRAS wild-type patients (A) and KRAS mutant patients (B), liver-limited versus other/multiple metastatic disease.

## Figures and Tables

**Table 1:** Young-elderly patients' features.

	Overall	*KRAS* wild-type	*KRAS* mutant
	Total no. (%)	Total no. (%)	Total no. (%)
No. of patients	28	13 (50)	13 (50)
Sex			
Male/female	14/14	6/7	8/5
Age, years			
Median	67	67	68
Range	65–73	65–73	66–73
WHO performance status			
0	25 (89)	12 (92)	11 (85)
1-2	3 (11)	1 (8)	2 (15)
CIRS stage			
Primary	2 (7)	—	2 (15)
Intermediate	26 (93)	13 (100)	11 (85)
Metastatic disease			
Metachronous	10 (36)	5 (38)	5 (38)
Synchronous	18 (64)	8 (62)	8 (62)
Primary tumor			
Colon	15 (54)	5 (38)	10 (77)
Rectum	13 (46)	8 (62)	3 (23)
Sites of metastases			
Liver	17 (61)	7 (54)	8 (62)
Lung	9 (32)	4 (31)	4 (31)
Lymph nodes	10 (36)	4 (31)	5 (38)
Local	7 (25)	4 (31)	3 (23)
Other	5 (18)	1 (8)	4 (31)
No. of involved sites			
1	14 (50)	8 (62)	5 (38)
≥2	14 (50)	5 (38)	8 (62)
Single metastatic sites			
Liver-limited	8 (29)	4 (31)	3 (23)
Other than liver	7 (25)	4 (31)	2 (15)
Lung	4 (14)	2 (15)	1 (8)
Lymph nodes	1 (4)	1 (8)	—
Local	2 (7)	1 (8)	1 (8)
Multiple metastatic sites	13 (46)	5 (38)	8 (62)
Liver metastases			
Single	8 (29)	5 (38)	3 (23)
Multiple	9 (32)	2 (15)	7 (54)
Previous adjuvant chemotherapy	6 (21)	3 (23)	2 (15)
FA/5-FU bolus	3 (11)	2 (15)	—
FOLFOX4	3 (11)	1 (8)	2 (15)
Previous radiotherapy	2 (7)	2 (8)	—
RT + CT (5-FU continuous infusion)	2 (7)	2 (8)	—
RT + CT (XELOX)	—	—	—

WHO: world health organization; CIRS: cumulative illness rating scale.

**Table 2:** Activity, efficacy, and effectiveness of FIr-B/FOx regimen in young-elderly patients according to *KRAS* genotype.

	All		*KRAS* wild-type		*KRAS* mutant	
	Intent-to-treat analysis		Intent-to-treat analysis		Intent-to-treat analysis	
	No.	%	No.	%	No.	%
Enrolled pts	28	100	13	100	13	100
Evaluable pts	28	100	13	100	13	100
Objective response	22	79 (CI ± 15)	12	92 (CI ± 15)	10	77 (CI ± 24)
Partial response	19	68	10	77	9	69
Complete response	3	11	2	15	1	8
Stable disease	1	4	—	—	1	8
Progressive disease	5	18	1	8	2	15
Median PFS, months	11		14		7	
Range	3–78+		4–78+		3–69+	
Progression events	23	82	10	77	11	85
Median OS, months	21		38		19	
Range	6–78+		8–78+		6–69+	
Deaths	19	68	9	69	8	61.5
Liver metastasectomies	5		3		2	
No/overall pts	5/28	18	3/13	23	2/13	15
No/Pts with liver metastases	5/17	29	3/7	43	2/8	25
No/Pts with L-L metastases	3/8	37.5	2/4	50	1/3	33
Pathologic complete responses	2	40	—	—	2	100

pts: patients; PFS: progression-free survival; OS: overall survival; L-L: liver-limited.

**Table 3:** Cumulative toxicity.

	Patients				Cycles			
Number	28				134			
NCI-CTC Grade	1	2	3	4	1	2	3	4
Nausea (%)	10 (36)	11 (39)	2 (7)	—	43 (32)	18 (13)	2 (1.5)	—
Vomiting (%)	7 (25)	3 (11)	2 (7)	—	15 (11)	5 (4)	2 (1.5)	—
Diarrhea (%)	12 (43)	8 (29)	6 (21)	—	48 (36)	15 (11)	7 (5)	—
Hypoalbuminemia (%)	1 (4)	1 (4)	—	—	1 (1)	1 (1)	—	—
Constipation (%)	12 (43)	—	—	—	16 (12)	—	—	—
Stomatitis/mucositis (%)	10 (36)	1 (4)	3 (11)	—	19 (14)	2 (1.5)	3 (2)	—
Erythema (%)	2 (7)	—	1 (4)	—	2 (1.5)	—	1 (1)	—
Asthenia (%)	9 (32)	11 (39)	3 (11)	—	30 (22)	23 (17)	3 (2)	—
Neurotoxicity (%)	21 (75)	4 (14)	—	—	72 (54)	4 (3)	—	—
Hypertension (%)	7 (25)	3 (11)	—	—	11 (8)	3 (2)	—	—
Hypotension (%)	1 (4)	—	—	—	1 (1)	—	—	—
Hematuria (%)	—	1 (4)	—	—	—	1 (1)	—	—
Gingival recession/gingivitis (%)	5 (18)	—	—	—	6 (4)	—	—	—
Rhinitis (%)	22 (78)	—	—	—	50 (37)	—	—	—
Epistaxis (%)	20 (71)	—	—	—	46 (34)	—	—	—
HFS (%)	—	—	—	—	—	—	—	—
Headache (%)	5 (18)	—	—	—	7 (5)	—	—	—
Hypokalemia (%)	2 (7)	—	—	—	2 (1.5)	—	—	—
Hypertransaminasemy (%)	3 (11)	1 (4)	—	1 (4)	5 (4)	2 (1.5)	—	1 (1)
Hyperpigmentation (%)	3 (11)	—	—	—	5 (4)	—	—	—
Fever without infection (%)	6 (21)	—	—	—	6 (4)	—	—	—
Alopecia (%)	3 (11)	7 (25)	3 (11)	—	8 (6)	14 (10)	7 (5)	—
Anemia (%)	3 (11)	2 (7)	—	—	7 (5)	2 (1.5)	—	—
Leucopenia (%)	10 (36)	11 (39)	—	—	34 (25)	17 (13)	—	—
Neutropenia (%)	4 (14)	11 (39)	3 (11)	—	25 (19)	25 (19)	3 (2)	—
Thrombocytopeny (%)	4 (14)	1 (4)	—	—	6 (4)	1 (1)	—	—

**Table 4:** Limiting toxicity syndromes (LTS): overall and in young-elderly patients.

	Overall		Young-elderly		Non-elderly	
	No.	%	No.	%	No.	%
Patients	67	100	28	42	39	58
Limiting toxicity syndromes (LTS)	32	48	13	46	19	49
LTS single site (LTS-ss)	10	15	2	7	8	21
LTS multiple sites (LTS-ms)	22	33	11	39	11	28
Single LT plus G2-3	15	22	9	32	6	15
Double LTs	7	10	2	7	5	13

LT: limiting toxicity; G: grade.
