# Supplementary material for: Effectiveness and Safety of Intensive Triplet Chemotherapy Plus Bevacizumab, FIr-B/FOx, in Young-Elderly Metastatic Colorectal Cancer Patients
Source: Biomed Res Int. 2013 Nov 6;2013:143273. doi: 10.1155/2013/143273 (PMC3838846; doi:10.1155/2013/143273)
Supplement: Supplementary file 1 — Among Supplementary material, Table 1 describes KRAS mutations detected; Table 2 describes received dose-intensities; Table 3 describes toxicities characterizing limiting toxicity syndromes in individual patients; Table 4 describes activity, efficacy and effectiveness of FIr-B/FOx regimen according to KRAS genotype and extension of metastatic disease; Figure 1 reports progression-free survival and overall survival of KRAS wild-type patients (A) and KRAS mutant patients (B), liver-limited versus other/multiple metastatic disease. [file 143273.f1.doc]

**Supplementary Materials Table 1: *KRAS* mutations**

| ***KRAS* mutant** | | | | | |
| --- | --- | --- | --- | --- | --- |
| **Exon** | **Codon** | **Hot spot site** | **Amino acid** | **No. of patients** | **%** |
| 2 | 12  13 | c.34 G > A  c.35 G > A  c.35 G > T  c.35 G > C  c.37_39 dupl  c.37  c.38 G > A | p.Gly12Ser  p.Gly12Asp  p.Gly12Val  p.Gly12Ala  p.Gly13dupl  -  p.Gly13Asp | **12**  -  8  3  1  **1**  -  -  1 | **46.1**  -  30.7  11.5  3.8  **3.8**  -  -  3.8 |

**Supplementary Materials Table 2: Dose-intensity**

|  |  | **All patients** | | **Young-elderly patients** | |
| --- | --- | --- | --- | --- | --- |
|  |  | **DI/cycle**  **mg/m^2^(or Kg)/w** | | **DI/cycle**  **mg/m^2^(or Kg)/w** | |
|  | **Projected DI**  **mg/m^2^(o Kg)/w** | **Median**  **(Range)** | **Received DI (%)** | **Median**  **(Range)** | **Received DI (%)** |
| **5-FU** | 1800 | 1440  (480-1800) | 80 | 1440  (480-1800) | 80 |
| **CPT-11** | 80 | 64.5  (25-80) | 80 | 64  (25-80) | 80 |
| **l-OXP** | 40 | 32  (8-40) | 80 | 32  (8-40) | 80 |
| **BEV** | 2.5 | 2.1  (0.4-2.5) | 84 | 2  (1-2.5) | 80 |
| Abbreviation: DI, dose-intensity; 5-FU, 5-Fluorouracil; CPT-11, Irinotecan; l-OXP, Oxaliplatin; BEV, Bevacizumab. | | | | | |

**Supplementary Materials Table 3: Limiting Toxicity Syndromes (LTS)**

| **Patients**  **#** | **Age**  **(years)** | **LT** | **Associated Toxicity** | |
| --- | --- | --- | --- | --- |
|  |  |  | **LT** | **G2-G3** |
| **1** | 71 | Diarrhea G3 | - | - |
| **2** | 70 | Asthenia G3 | - | - |
| **3** | 68 | Diarrhea G3 | - | Vomiting G3 |
| **4** | 67 | Diarrhea G3 | - | Nausea G3  Asthenia G2 |
| **5** | 67 | Diarrhea G3 | - | Vomiting G2 |
| **6** | 65 | Diarrhea G3 | - | Epistaxis G2 |
| **7** | 66 | Diarrhea G3 | - | Stomatitis/mucositis G2  Asthenia G2 |
| **8** | 67 | Stomatitis/mucositis G3 | - | Asthenia G2 |
| **9** | 66 | Neurotoxicity G2 | - | Asthenia G2  Diarrhea G2 |
| **10** | 73 | Neutropenia G2 for > 2 weeks | - | Nausea G2 |
| **11** | 66 | Hypertransaminasemy G4 | - | Diarrhea G2  Nausea G2  Anemia G2 |
| **12** | 71 | Diarrhea G3 | Stomatitis/mucositis G3 | Hypoalbuminemia G2 |
| **13** | 66 | Stomatitis/mucositis G3 | Erythema G3 | - |
| Abbreviation: LT, limiting toxicity; G, grade. | | | | |

**Supplementary Materials Table 4: Activity, efficacy and effectiveness of FIr-B/FOx regimen according to *KRAS* genotype and extension of metastatic disease**

|  | **All** | | | ***KRAS* wild-type** | | | ***KRAS* mutant** | |
| --- | --- | --- | --- | --- | --- | --- | --- | --- |
|  | **L-L** | **O/MM** | | **L-L** | **O/MM** | | **L-L** | **O/MM** |
| **Evaluable pts** | 7 | 19 | | 4 | 9 | | 3 | 10 |
| **Objective Response**  **(%; C.I.)**  Partial Response  Complete Response | 6  (86; ± 28)  3  3 (43) | 16  (84; ± 17)  16  - | | 4  (100)  2  2 (50) | 8  (89; ± 22)  8  - | | 2  (67; ± 65)  1  1 (33) | 8  (80; ± 26)  8  - |
| **Stable Disease** | - | 1 | | - | - | | - | 1 |
| **Progressive Disease** | 1 | 2 | | - | 1 | | 1 | 1 |
| **Liver metastasectomies N. (%)**  **Pathologic complete responses** | 3 (43)  1 (14) | 2 (10.5)  1 (5) | | 2 (50)  - | 1 (11)  - | | 1 (33)  1 (33) | 1 (10)  1 (10) |
| **Overall activity* (N.; %)** | 6 (86) | 2 (10.5) | | 4 (100) | 1 (11) | | 2 (67) | 1 (10) |
| **Median PFS, months**  Range  Progression events | 30  3-78+  4 | 11  4-18  17 | | n.r.  3-69+  2 | 12  4-16  8 | | 17  3-69+  2 | 7  4-18  9 |
| p | 0.011 | | | 0.058 | | | 0.295 | |
| **Median OS, months**  Range  Deaths | n.r.  20-78+  2 | | 19  6-59  15 | n.r.  33-78+  1 | | 31  8-59  8 | n.r.  20-69+  1 | 19  4-47+  7 |
| p | 0.005 | | | 0.035 | | | 0.106 | |
| Abbreviation: pts, patients; *, clinical complete response + metastasectomies; L-L, liver-limited; O/MM, other/multiple metastatic site; PFS, progression-free survival; OS, overall survival. | | | | | | | | |

**Supplementary Materials Figure 1: Kaplan-Meier survival estimate**

A, L-L versus O/MM, *KRAS* wild-type; B, L-L versus O/MM, *KRAS* mutant; 1, PFS; 2, OS.


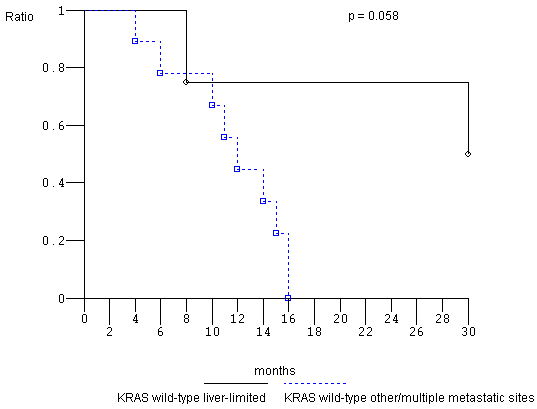

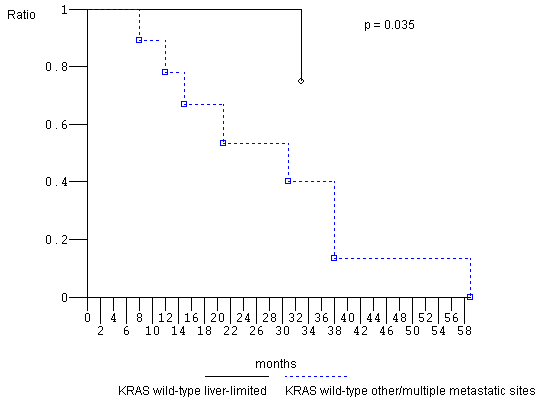


**(A1) (A2)**


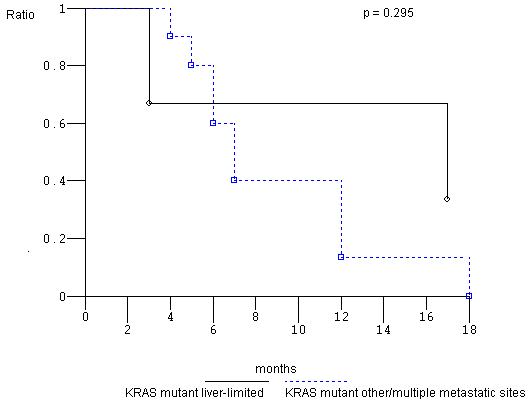

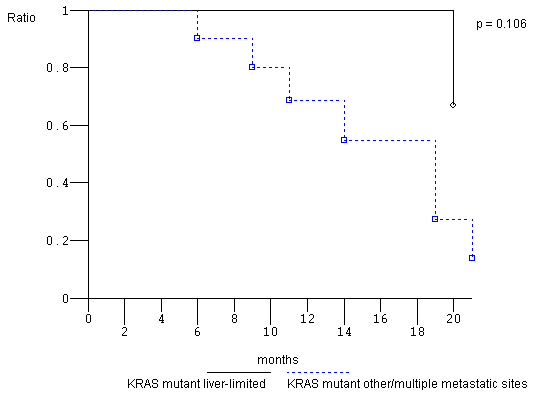


**(B1) (B2)**
